# Effect of buried vs. exposed Kirschner wire osteosynthesis on phalangeal, metacarpal and distal radial fractures: A systematic review and meta-analysis

**DOI:** 10.1186/s42836-020-0021-5

**Published:** 2020-02-03

**Authors:** Long Chen, Yuanzheng Wang, Senlei Li, Rui Luo, Wei Zhou, Yankun Li, Gong Zhang, Xianghe Li, Cheng Wang, Chao Hao, Lingchao Kong, Yangyang Li, Li Sun

**Affiliations:** 1 Department of Orthopedics, Guizhou Provincial People’s Hospital, Guiyang, 550000 Guizhou China; 2 Graduate School of Clinical Medicine, Zunyi Medical University, Zunyi, 563006 Guizhou China; 3 School of Clinical Medicine, Guizhou Medical University, Guiyang, 550000 Guizhou China

**Keywords:** Buried kirschner wire, Exposed kirschner wire, Osteosynthesis, Phalangeal fractures, Metacarpal fractures, Distal radial fractures

## Abstract

**Background:**

During Kirschner wire osteosynthesis for phalangeal, metacarpal and distal radial fractures, a key clinical decision is whether to leave a Kirschner wire exposed or bury it beneath the skin. Therefore, we conducted a meta-analysis to evaluate the clinical effect of buried and exposed Kirschner wire osteosynthesis on phalangeal, metacarpal and distal radial fractures.

**Methods:**

PubMed, EMBASE and Cochrane Central Register of Controlled Trials (CENTRAL) databases were searched and clinical trials that evaluated buried and exposed Kirschner wire osteosynthesis for phalangeal, metacarpal and distal radial fractures were identified. Methodological qualities of studies were assessed by using the Cochrane Collaboration tool and Newcastle-Ottawa Scale. Publication bias was detected using Begg’s test and Egger’s test. Sensitivity analyses were performed by excluding one study at a time to determine whether overall results were reliable.

**Results:**

Two randomized controlled trials (RCT) and five case-controlled studies involving 1446 patients were included in the analysis. Sensitivity analyses indicated that the results of each study were statistically robust. Begg’s test or Egger’s test revealed no significant publication bias. Our meta-analysis indicated that exposed Kirschner wire osteosynthesis had a significantly higher infection rate than its buried counterpart. Additionally, buried Kirschner wire osteosynthesis resulted in a significantly higher rate of Kirschner wire removal in operating room than the exposed one. Our meta-analysis exhibited no statistical differences between the two procedures in the rate of early pin removal.

**Conclusions:**

Meta-analysis of available RCTs and case-controlled studies demonstrated that buried Kirschner wire osteosynthesis had a lower infection rate but tended to lead to more frequent Kirschner wire removal in the operating room as compared to its exposed counterpart.

**Trial registration:**

PROSPERO (CRD42018105249).

## Introduction

Distal radial, phalangeal and metacarpal fractures are the first, second, and third common upper extremity fractures, with their incidences roughly standing at 16.2, 12.5 and 8.4 per 10,000 person-years, respectively [1]. Kirschner wire osteosynthesis is the most common technique for bone fixation in unstable distal radius, phalange and metacarpal fractures because it is minimally invasive, relatively inexpensive and versatile [2, 3].

With Kirschner wire osteosynthesis, a critical clinical decision is whether to leave a Kirschner wire exposed or to bury it beneath the skin. According to a survey conducted in the United Kingdom [4], hand surgeons preferred to leave Kirschner wires exposed due to the ease of removal. However, patients are concerned more about wire-related problems, such as pin infection, recovery and pain. The pin infection could be disastrous for Kirschner wire osteosynthesis and can lead to failure of fixation, malunion and osteomyelitis. Hargreaves et al. [5] and Ridley et al. [6] previously reported that exposed Kirschner wires had a significantly greater infection rate than buried Kirschner wires for phalangeal, metacarpal and distal radius fractures. On the contrary, Mcquail et al. [7] found that buried Kirschner wires resulted in a higher rate of infection compared to exposed Kirschner wires for distal radius fracture. In addition, Koç et al. [8] and Terndrup et al. [9] reported that there was no statistically significant difference in the rate of infection between buried and exposed Kirschner wires. Although a recent systematic review [10] qualitatively described the infection rate of buried versus exposed Kirschner wires in the upper extremity, debate remains because of lack of quantitative analysis.

This meta-analysis systematically reviewed the randomized controlled trials (RCT) and case-controlled studies regarding Kirschner wire osteosynthesis for phalangeal, metacarpal and distal radial fractures. The objective was to compare the clinical effects of buried and exposed Kirschner wire osteosynthesis for phalangeal, metacarpal and distal radial fractures by using quantitative methods.

## Materials and methods

This study was performed in accordance with the criteria of the Preferred Reporting Items for Systematic Reviews and Meta-Analyses (PRISMA) [11]. We also registered this systematic review and meta-analysis in PROSPERO (CRD42018105249).

### Data sources and searches

PubMed, EMBASE and Cochrane Central Register of Controlled Trials (CENTRAL) databases were searched for relevant clinical trials. We performed this search in July 2018. To identify unpublished studies, we also searched orthopedic conferences Websites, ClinicalTrials.gov and International Clinical Trials Registry Platform. According to the principles of PICOS (population, intervention, comparison, outcome and study design), the search strategy was as follows: (phalangeal fractures or metacarpal fractures or distal radial fractures) and ((exposed Kirschner wire or percutaneous Kirschner wire) and buried Kirschner wire) and (randomized controlled trials or case-controlled trials).

### Inclusion and exclusion criteria

Inclusion criteria for literature were as follows: (1) target population: patients with a clinical diagnosis of phalangeal, metacarpal and distal radial fractures; (2) intervention: using either exposed Kirschner wire or buried Kirschner wire osteosynthesis; (3) methodological criteria: RCTs and case-controlled studies.

Exclusion criteria were as follows: (1) target population: patients with fractures of positions excepting phalange, metacarpal and distal radius; (2) intervention: fixations with other implants (like screws and plates); (3) methodological criteria: case reports.

### Outcome assessment

The primary outcome measure was infection rate and the secondary outcome measures included the rate of Kirschner wire removal in operating room and the rate of early pin removal.

### Data extraction and quality assessment

All data of the qualified studies were extracted by two independent reviewers. Data about the country, study design, patient sample size, different interventions and length of follow-up of the included studies were collected. Additionally, clinical data that involved infection rate, Kirschner wire removal in operating room and early pin removal were extracted whenever available.

The Cochrane Collaboration tool for assessing risk of bias [12] was employed to assess the quality of RCTs against the following criteria: randomization sequence generation, allocation concealment, assessment of selection bias, level of blinding, assessment for performance bias and detection bias, incomplete outcome data and selective reporting. The Newcastle-Ottawa Scale [13] was used to assess the quality of case-controlled trials. Newcastle-Ottawa Scale score was measured on a 9-point scale against the criteria listed in Table 1.

**Table 1:** The Newcastle-Ottawa Scale (NOS) for assessing the quality of case controlled studies in meta-analyses

Selection	Comparability	Exposure
1) Is the case definition adequate?	1) Comparability of cases and controls on the basis of the design or analysis	1) Ascertainment of exposure
a) yes, with independent validation*		a) secure record (e.g.: surgical records)*
b) yes, e.g. record linkage or based on self reports		b) structured interview where blind to case/control status*
c) no description	a) study controls for __ (Select	
2) Representativeness of the cases	the most important factor.)*	c) interview not blinded to case/control status
a) consecutive or obviously representative series	b) study controls for any additional factor (This criteria could be modified to indicate specific control for a second important factor.)*	
of cases*		d) written self report or medical record only
b) potential for selection biases or not stated		
3) Selection of Controls		e) no description
a) community controls*		2) Same method of ascertainment for cases and controls
b) hospital controls		
c) no description		a) yes*
		b) no
4) Definition of Controls		3) Non-Response rate
a) no history of disease (endpoint)*		a) same rate for both groups*
b) no description of source		b) non respondents described
		c) rate different and no designation

Note: A study can be awarded a maximum of one star for each numbered item within the Selection and Exposure categories. A maximum of two stars can be given for Comparability". The "*" means one star

From GA. W, B. S, D. OC, et al. The Newcastle-Ottawa Scale (NOS) for assessing the quality of nonrandomised studies in meta-analyses

### Data synthesis and analysis

The statistical calculation of this meta-analysis was performed using the STATA software package (Vision 12.0). For each study, the odds ratio (OR) with 95% confidence intervals (CIs) was calculated for dichotomous data; mean differences (MD) with 95% CIs were computed for continuous data. A *P* value of 0.05 or less was considered statistically significant. Heterogeneity was detected using the chi-squared and I^2^ statistics. When I^2^ was greater than 50% and *P* value less than 0.05, heterogeneity was considered significant across the included studies. A fixed effects model was employed for analysis with no evidence of significant heterogeneity, otherwise, a random effect model was used in the analysis with evidence of significant heterogeneity. Egger’s and Begg’s tests were conducted of the meta-analysis to assess publication bias, and *P* values less than 0.05 for each test were interpreted as evidence of publication bias [14, 15]. Sensitivity analyses that excluded one study at a time were performed to determine whether results were reliable [16]. In addition, for outcomes regarding infection rate and Kirschner wire removal in the operating room, we conducted both publication bias and sensitivity analysis. We did not perform the publication bias and sensitivity analysis for the outcome of early pin removal due to insufficient data from only two studies included in this analysis.

## Results

### Study selection

**Fig. 1:**
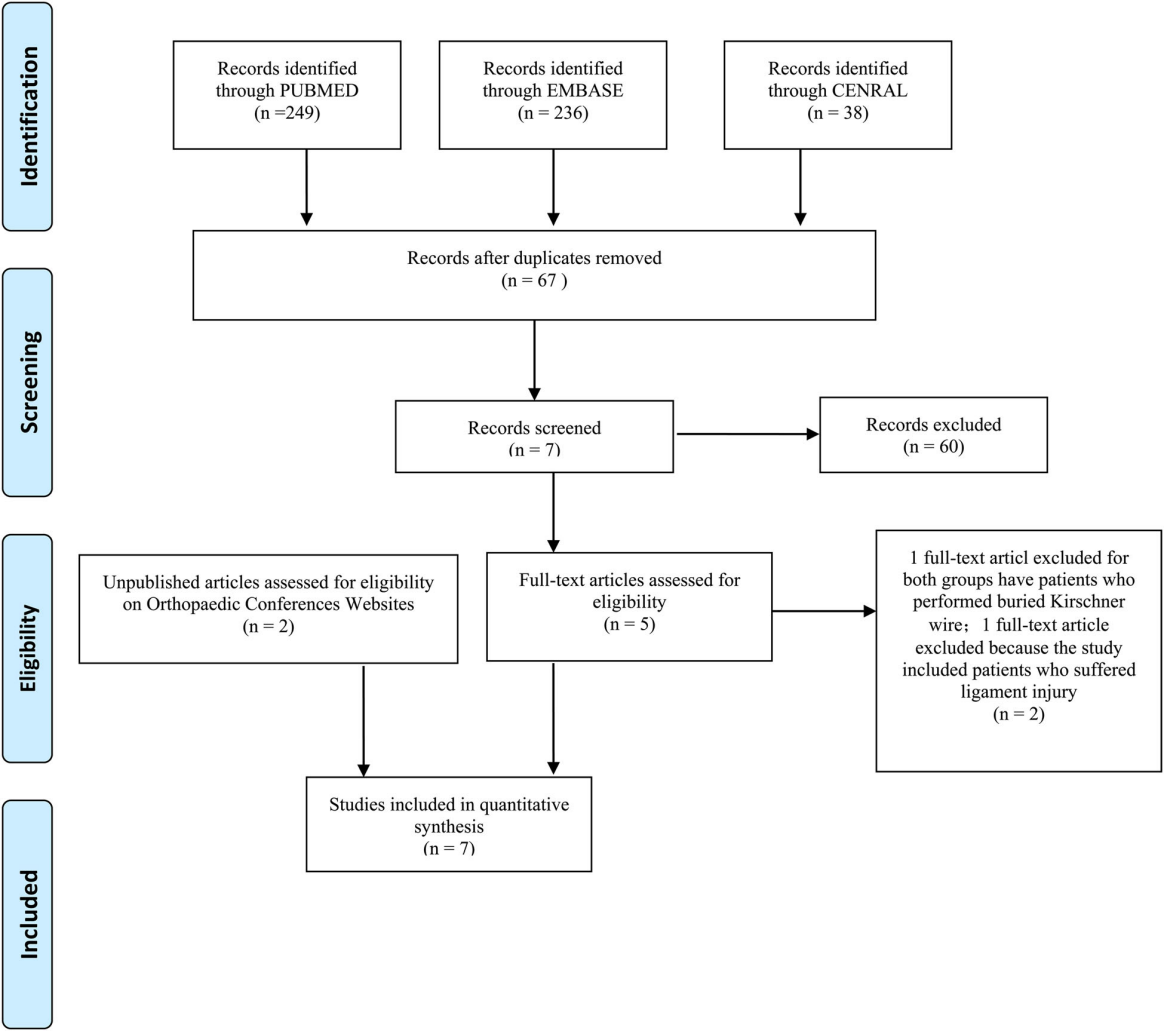
Flow chart of selection of studies for inclusion in meta-analysis

The study selection was performed according to the PRISMA flow diagram (Fig. 1). A total of 523 studies (including 249, 236 and 38 from PubMed, EMBASE and CENTRAL, respectively) were originally included in this study. After each of two reviewers read the full-text, one study was excluded for the study included patients who received both exposed and buried Kirschner wire osteosynthesis [17], another was excluded because the study included patients who suffered ligament injury [8]. One RCT [5] and four case-controlled trials [6, 9, 18, 19] were finally included in this meta-analysis. After searching orthopedic conferences Websites, ClinicalTrials.gov and International Clinical Trials Registry Platform, two unpublished conference studies (one randomized controlled trial and one case-controlled trial) [7, 20] were also included in the final quantitative analysis.

### Characteristics and quality of the included studies

**Fig. 2:**
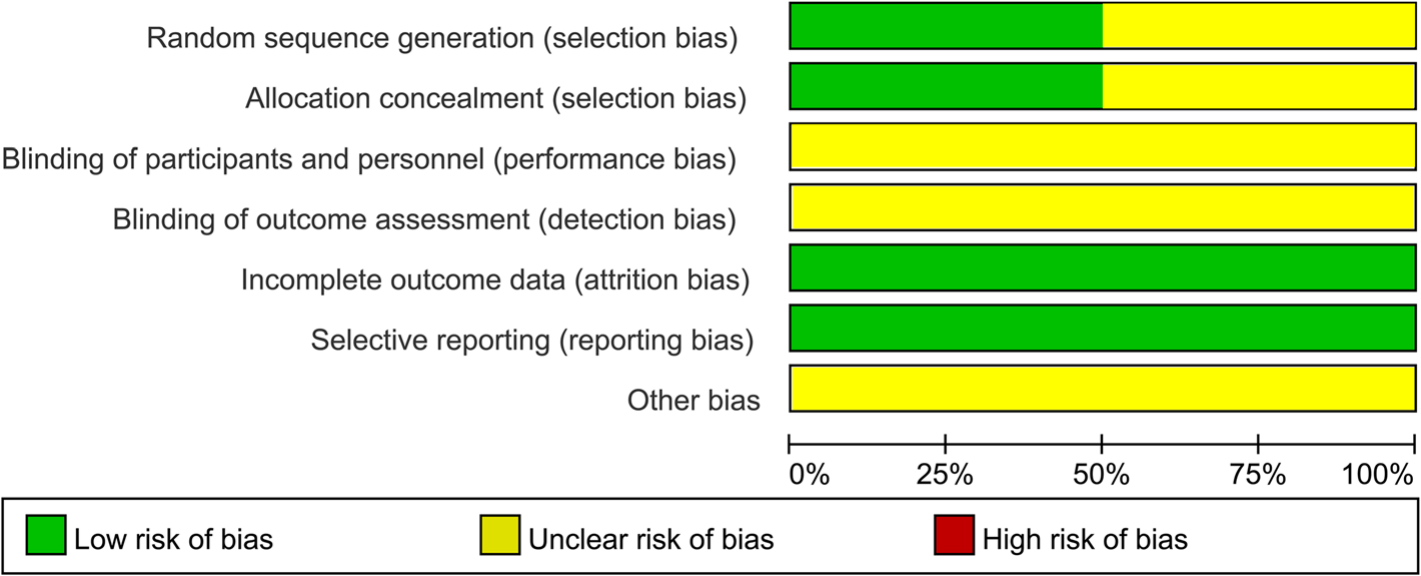
Risk of bias graph: review authors’ judgements about each risk of bias item presented as percentages across all included randomized controlled trials

Seven studies involving a total of 1446 patients were included in the analysis. Study characteristics (country, interventions, sample size, length of follow-up, study design) are presented in Table 2. Of the two RCTs analyzed [5, 20], the Cochrane Collaboration tool indicated that one trial [5] used adequate randomization and allocation concealment. Two included RCTs [5, 20] were also shown to be free of selective reporting and incomplete outcome data (Fig. 2). As assessed by Newcastle-Ottawa Scale, two case-controlled studies [6, 9] were awarded a score of eight points, while the other three studies [7, 18, 19] received a score of seven points (Table 3).

**Table 2:** Characteristics of included studies comparing buried versus exposed Kirschner wire osteosynthesis for phalangeal, metacarpal and distal radial fractures

Study	Country	Interventions	Sample size	Follow-up (week)	Study design	For analysis
AI-Qattan 2008	Saudi Arabia	Exposed vs Buried	40/38	14	Retrospective comparative	Infection rate
Hargreaves 2004 [5]	UK	Exposed vs Buried	29/27	6	RCT	Infection rate; Kirschner wire removal in operating room; Early pin removal
Mcquail 2015 [7]	Ireland	Exposed vs Buried	33/28	6	Retrospective comparative	Infection rate
Rafique 2006 [19]	Pakistan	Exposed vs Buried	30/30	4	Retrospective comparative	Infection rate
Ridley 2017 [6]	USA	Exposed vs Buried	488/207	–	Retrospective comparative	Infection rate; Early pin removal
Terndrup 2018 [9]	Denmark	Exposed vs Buried	107/337	13	Retrospective comparative	Infection rate; Kirschner wire removal in operating room
Waheed 2004 [20]	Ireland	Exposed vs Buried	27/25	5.8	RCT	Kirschner wire removal in operating room

Note: *RCT* Randomized controlled trial

**Table 3:** Quality assessment of case controlled studies comparing buried versus exposed Kirschner wire osteosynthesis for phalangeal, metacarpal and distal radial fractures using Newcastle-Ottawa Scale

Author group	Selection				Comparability		Exposure	
	Adequae case definitin	Representativeness of the cases	Selection of Controls	Definition of Controls	Comparability of cases and controls	Ascertainm-ent of exposure	Same method of ascertainment	Non Response rate
AI-Qattan 2008	1	1	–	1	1	1	1	1
Mcquail 2015 [7]	–	1	1	1	1	1	1	1
Rafique 2006 [19]	–	1	1	1	1	1	1	1
Ridley 2017 [6]	1	1	1	1	1	1	1	1
Terndrup 2018 [9]	1	1	1	1	1	1	1	1

### Infection rate

**Fig. 3:**
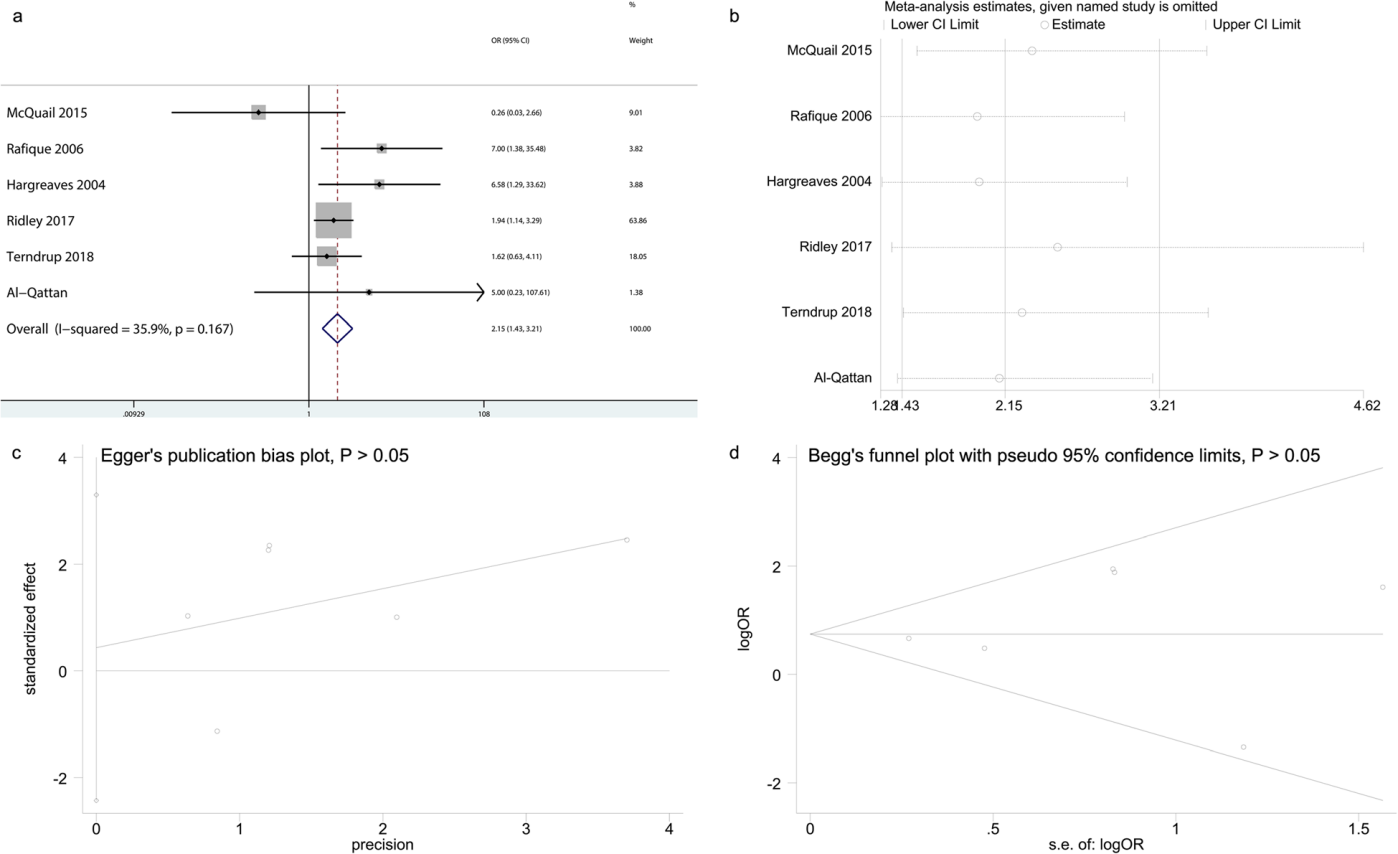
The results of data synthesis for infection rate. **a**: Forest plot showing the infection rate comparing buried versus exposed Kirschner wire osteosynthesis for phalangeal, metacarpal and distal radial fractures; **b**: Sensitivity analyses for determining the reliability of results; **c**: Egger’s publication bias plot; **d**: Begg’s funnel plot

Regarding the infection rate, six trials [5–7, 9, 18, 19] were included in this meta-analysis. A total of 727 patients underwent exposed Kirschner wire osteosynthesis and 667 received buried Kirschner wire osteosynthesis. This meta-analysis demonstrated that exposed Kirschner wire osteosynthesis resulted in a significantly higher infection rate than buried Kirschner wire osteosynthesis (OR: 2.15, 95% CI: 1.43–3.21, *P* = 0.0001, I^2^ = 35.9%; Fig. 3a). Sensitivity analysis was performed to investigate the influence of each individual study on the pooled OR by excluding one study from analysis at a time. The estimated results revealed that no single study significantly affected the pooled OR in terms of infection rate (Fig. 3b), demonstrating the results were statistically robust. Additionally, results of Egger’s and Begg’s test revealed that the meta-analysis of infection rate had no significant publication bias (Fig. 3c and d).

### Kirschner wire removal in operating room

**Fig. 4:**
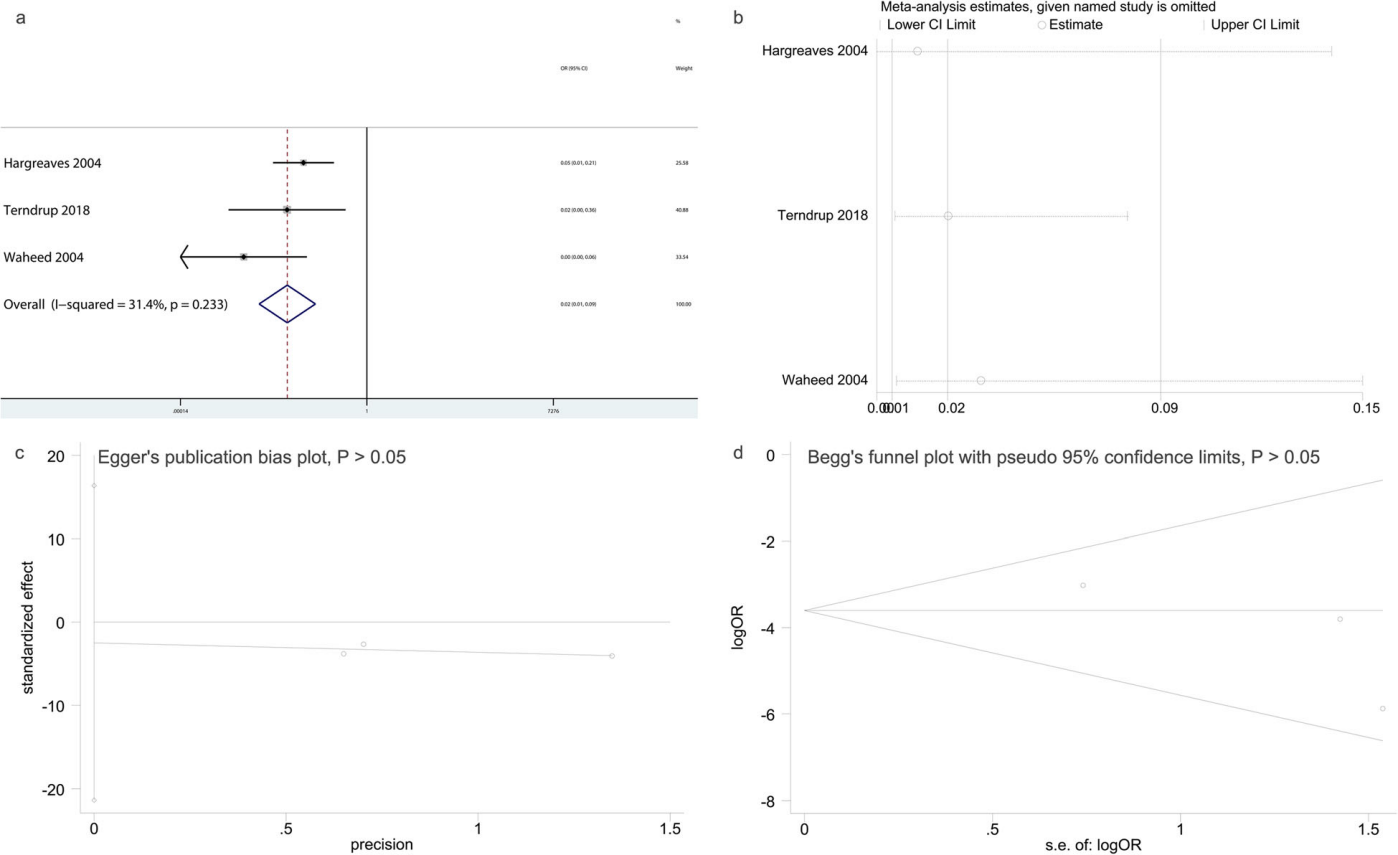
The results of data synthesis for Kirschner wire removal in operating room. **a**: Forest plot showing the rate of Kirschner wire removal in operating room comparing buried versus exposed kirschner wire osteosynthesis for phalangeal, metacarpal and distal radial fractures; **b**: Sensitivity analyses for determining the reliability of results; **c**: Egger’s publication bias plot; **d**: Begg’s funnel plot

As to the Kirschner wire removal in operating room, three studies [5, 9, 20] were included in this meta-analysis. A total of 163 patients underwent exposed Kirschner wire osteosynthesis and 389 were treated by buried Kirschner wire osteosynthesis. We demonstrated that buried Kirschner wire osteosynthesis resulted in a significantly higher rate of Kirschner wire removal in the operating room than exposed Kirschner wire osteosynthesis (OR: 0.02, 95% CI: 0.01–0.09, *P* = 0.0001, I^2^ = 31.4%; Fig. 4a). Sensitivity analysis also revealed that no single study significantly affected the pooled OR in Kirschner wire removal in operating room (Fig. 4b), demonstrating the results were statistically robust. Results of Egger’s and Begg’s test demonstrated that the meta-analysis of Kirschner wire removal in operating room had no significant publication bias (Fig. 4c and d).

### Early pin removal

**Fig. 5:**
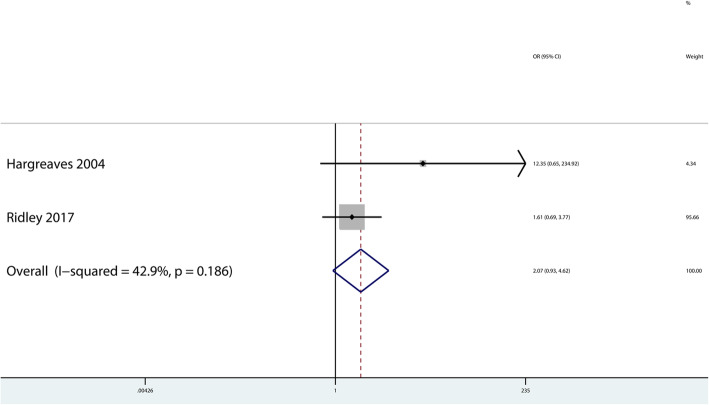
Forest plot showing the rate of early pin removal comparing buried versus exposed Kirschner wire osteosynthesis for phalangeal, metacarpal and distal radial fractures

As to the early pin removal, two studies [5, 6] were included in this meta-analysis. A total of 517 patients underwent exposed Kirschner wire osteosynthesis and 234 underwent buried Kirschner wire osteosynthesis. This meta-analysis demonstrated no statistically significant difference in early pin removal between exposed and buried Kirschner wire osteosynthesis (OR: 2.07, 95% CI: 0.93–4.62, *P* = 0.074, I^2^ = 42.9%; Fig. 5). Due to the small number of trials included, we did not analyze publication bias or execute sensitivity analysis for the outcome of early pin removal.

## Discussion

This meta-analysis included data from two RCTs and five case-controlled trials involving 1446 patients with hand and wrist fractures. Our meta-analysis indicated that exposed Kirschner wire osteosynthesis resulted in a significantly higher infection rate than buried Kirschner wire osteosynthesis. Buried Kirschner wire osteosynthesis led to in a significantly higher rate of Kirschner wire removal in the operating room than exposed Kirschner wire osteosynthesis. Our meta-analysis also indicated that there were no statistical differences between the two procedures in terms of early pin removal rate. The results of sensitivity analyses, Egger’s and Begg’s test demonstrated that the results of this meta-analysis were statistically robust and had no significant publication bias.

There are some particular strengths to this meta-analysis: (1) it is the first study that quantitatively compared the clinical effect of buried versus exposed Kirschner wire osteosynthesis for phalangeal, metacarpal and distal radial fractures. (2) The broad and extensive search strategy, covering database and conference websites could minimize the possibility of publication bias and Egger’s and Begg’s test also demonstrated that there was no significant publication bias in this meta-analysis. (3) We performed this meta-analysis by using common methods to allow for reproducible research selection and inclusion.

However, this analysis has several limitations which should be noted. Firstly, we included both RCTs and case-controlled trials in this analysis, which might have reduced the significance of the individual condition. Secondly, due to the small number of trials in the analysis of early pin removal, accuracy might have been impaired. Finally, the features of included studies, such as type of fracture, number of wires and level of surgeon’s proficiency might be potential biases that affect the outcomes of our study.

A retrospective study found that the incidence of pin track infection was 7% in 1213 patients treated with Kirschner wire fixation for hand and wrist fractures [2]. Another study about 189 patients undergoing exposed or buried Kirschner wire procedures involving the hand and wrist, identified 19 patients (10%) who developed pin infection [3]. However, the infection rate between exposed and buried Kirschner wire osteosynthesis for hand and wrist fracture was controversial [5–9]. Our meta-analysis exhibited that exposed Kirschner wire osteosynthesis resulted in a significantly higher infection rate than buried Kirschner wire osteosynthesis. Early pin removal might be performed when the patient developed postoperative infection. However, we found that there was no statistical difference in the the rates of early pin removal between the two procedures.

Kirschner wires are removed after a fracture has healed. In clinical practice, exposed wires are easily removed in the out-patient setting, thus avoiding the need for added general anesthetics, operating time and cost associated with removal of the buried Kirschner wires [21]. That explains why the hand surgeons prefer to leave Kirschner wires exposed. In our meta-analysis, we also found that buried Kirschner wire osteosynthesis resulted in a significantly higher rate of Kirschner wire removal in operating room than exposed Kirschner wire osteosynthesis. Moreover, a cost analysis performed by Koç et al. demonstrated that burying Kirschner wires incurred an extra cost of £235.51 per patient against £90.80 per patient for exposed Kirschner wires [8]. For this reason, buried Kirschner wire osteosynthesis had the higher rate of Kirschner wire removal in operating room and cost more than exposed Kirschner wire osteosynthesis.

Although the decision whether to leave the Kirschner wire exposed or to bury it beneath the skin may be influenced by multiple factors (such as type of fractures, soft tissue condition and the patient’s age), our study still provided useful information about infection rate, Kirschner wire removal in operating room and early pin removal of buried versus exposed Kirschner wire osteosynthesis for phalangeal, metacarpal and distal radial fractures. Even though it may incur higher costs, we still recommend that Kirschner wire should be buried, because it is safe and has lower infection rate.

## Conclusions

In summary, our meta-analysis demonstrated that buried Kirschner wire osteosynthesis resulted in a lower infection rate, but led to more frequent Kirschner wire removal in the operating theater than exposed Kirschner wire osteosynthesis procedures.

## Data Availability

Not applicable.

## Abbreviations

CENTRAL: Cochrane Central Register of Controlled Trials
CI: Confidence interval
MDs: Mean differences
OR: Odds ratio
PRISMA: Preferred Reporting Items for Systematic Reviews and Meta-Analyses
RCT: Randomized controlled trials
